# The Natural Environment of Physical Activity and Perceived Stress: The Mediating Role of Specific Recovery Experiences

**DOI:** 10.3389/fspor.2021.706467

**Published:** 2021-08-16

**Authors:** Julia Schmid, Lars Imbach, Sandra Klaperski, Gorden Sudeck

**Affiliations:** ^1^Department of Sport Psychology and Research Methods, Institute of Sport Science, University of Bern, Bern, Switzerland; ^2^Department of Psychology, Faculty of Social and Behavioural Sciences, University of Amsterdam, Amsterdam, Netherlands; ^3^Department of Education & Health Research, Institute of Sport Science, University of Tübingen, Tübingen, Germany

**Keywords:** green exercise, stress regulation, ecological momentary assessment, restorative experiences, natural environment

## Abstract

**Objective:** The purpose of this study was to investigate a potential psychological mechanism of green exercise on perceived stress. More precisely, it was analyzed whether the relationship between the natural environment of physical activity and perceived stress was mediated by recovery experiences, namely by psychological detachment and relaxation. An ecological momentary assessment approach was used, meaning that specific recovery experiences were assessed directly in real-life situations and multiple times.

**Materials and methods:** Thirty five women and 27 men took part in the ecological momentary assessment study over seven days (*M*_age_ = 32.30 years, *SD* = 10.23, 53% had a degree from a university or a university of applied science). If participants were involved in PA lasting at least 10 min on a given day, they had to answer questionnaires on the smartphone both prior to the activity and immediately afterwards. Perceived naturalness, psychological detachment and relaxation were assessed after physical activity events, whereas perceived stress was measured before and after each physical activity event. A two-level mediation analysis was conducted. The direct and indirect effect of perceived naturalness on perceived stress after engagement in physical activity was analyzed on the within- and between-person levels.

**Results and conclusion:** Results showed that the relaxation as a recovery experience served as mediator between perceived naturalness and perceived stress after engagement in physical activity, but only on a within-person level. This means that the more natural a given individual appraised the physical activity environment, the more relaxed he or she felt during physical activity (β = *0.3*22, *p* < 0.0005). Furthermore the more relaxed the individual was, the less stress he or she perceived after exercising (β = −0.221, *p* < 0.0005). The psychological detachment as a recovery experience in contrast, did not serve as mediator, neither at the within- and the between-person level. Considering the indirect effect of perceived naturalness on perceived stress and the importance of relaxation experiences, current findings suggest that research should put greater emphasis on examining the specific psychological mechanisms of green exercise to make even better use of its beneficial effects in the future.

## Introduction

Work is consistently being reported to be one of the main sources of stress in daily life (American Psychological Association, 2019). In Switzerland, 29.6% of all employees felt stressed at work in the year 2020. Just like in other Western nations, this number has increased over the last six years (Galliker et al., 2020; Health Safety Executive, 2020). This development is worrying, as numerous meta-analyses and reviews show that a high number of job stressors (e.g., high workload, intrapersonal conflicts) often lead to illnesses and poor mental well-being, particularly when job stressors persist over longer periods of time (Crawford et al., 2010; Nixon et al., 2011; Law et al., 2020). These negative impacts of job stressors can be reduced with recovery activities during non-working time (Sonnentag, 2018a). Physical activity (PA) has been shown to be a very effective recovery activity (see for a summary Sonnentag, 2018b), with about 60% of adults reporting that they exercise and do sports to reduce stress (Schmid et al., 2018). However, little is known about which settings or what types of activities are particularly effective (Klaperski et al., 2019).

In the past decades, there has been growing research interest in PA in natural environment, so-called “green exercise.” It is hypothesized that direct exposure to nature (e.g., in parks, forests or by lakes) increases the positive effects PA has on mental well-being (Pretty, 2004). While a narrative review from 2011 found that green exercise produced a more favorable impact on mental well-being when compared to indoor exercise (Thompson Coon et al., 2011), more recent reviews show little or no added value (Lahart et al., 2019; Mnich et al., 2019). Overall, existing evidence is still limited and characterized by the heterogeneity of studies. Authors have identified several research gaps that need to be addressed.

A first important issue that future research should investigate are the underlying mechanisms of green exercise (Mnich et al., 2019). If we know which factors may promote mental well-being, they can be better targeted in studies and health promotion programs. Various theories in the field of environmental psychology have been proposed to explain the potential effect of nature, with the Stress Recovery Theory (SRT; Ulrich, 1983) and the Attention Restoration Theory (ART; Kaplan and Kaplan, 1989) being particularly prominent. SRT assumes that humans are programmed to react emotionally positively to natural settings and elements because these settings and elements are evolutionarily associated with safety and survival. This in turn, should reduce or buffer stress. Whereas, SRT focusses on affective benefits that may derive from nature, ART focusses on cognitive benefits. ART assumes that nature provides stimuli that attract involuntary, automatic attention. This effortless mode of attention allows individuals to recover from mental fatigue which occurs after performing cognitive tasks that require directed attention. Both theories highlight the negative impact of stress and suggest that nature-rich places are more likely to facilitate restoration or recovery experiences than built or urban environment (Joye and Dewitte, 2018). However, the theories differ with regard to the specific experiences they consider to be key in positively affecting mental well-being. Kaplan and Kaplan (1989) suggest, as asserted by their ART, that natural environments need to create a sense of “being away” to have a recovery effect. Concerning the SRT, Ulrich et al. (1991, p. 226) assume that natural settings generate a “mild, eyes-open form of relaxation response or wakeful, meditation-like state,” which leads to their stress reducing effect. While there is only limited evidence on the role of these specific experiences of “being away” and relaxation in green exercise research, there has been a lot of research examining these concepts in the field of occupational health psychology: Sonnentag and Fritz (2007) identified psychological detachment and relaxation as two crucial experiences during off-job time that promote recovery from daily stressors at work.

Psychological detachment refers to the experience of “switching off.” It is characterized by being physically away from the workplace (i.e., one does not engage in any work-related activities) and by mentally disengaging from work during leisure time (i.e., one does not think about work in one's free time). In contrast, relaxation refers to the positively-toned state of low activation (Sonnentag and Fritz, 2007). Meta-analytic evidence shows that both recovery experiences are associated with better mental well-being on a day-to-day level (Bennett et al., 2018). Consequently, it seems reasonable to hypothesize that psychological detachment and relaxation mediate the potential effect of green exercise on mental well-being by reducing the effects of stress. Yet only a few studies provide insights into this research topic to this day. For instance, Feuerhahn et al. (2014) demonstrated that the relation between PA and positive affect in the evening was explained by psychological detachment. However, in this study the natural environment of PA was not considered. Furthermore, Korpela et al. (2014) showed that the link between nature-based outdoor activities and well-being was mediated by a global measure of recovery experiences. Yet again, these research findings have limited transferability, because the study did not focus specifically on PA and detachment or relaxation. To the best of our knowledge, no study has yet examined the relationship between PA in a natural environment, psychological detachment, relaxation and perceived stress.

A second issue in existing green exercise research is that studies on short-term effects and mechanisms often use only a single bout of exercise, mainly being walking or running, and mostly in artificial laboratory conditions (Lahart et al., 2019; Mnich et al., 2019). Here, an ecological momentary assessment approach (EMA) may offer promising new insights. In EMA studies, features are measured immediately and multiple times in real-life situations, often by using smartphones (Dunton, 2017). Measuring features in real-life situations has the advantage that recovery experiences can be recorded as close as possible to the actual activity under natural conditions. The repeated measurements furthermore allow for the study of both intra-individual variations (within-person level) and inter-individual variations (between-person level). This is important, as the mechanisms of green exercise on mental well-being can be different depending on which level is being examined. For example, a negative association between psychological detachment and perceived stress after PA on the within-person level would mean that a given individual in different PA bouts experienced less stress if the activity allowed for distance from work. In contrast, the same negative link on the between-level would indicate that individuals who experienced a higher level of psychological detachment during PA on average would report lower stress levels afterwards. If the decomposition of within-person and between-person effects were ignored and only between-person effects were considered, an improper generalization of between-person effects could occur. The between-person relationship would be inappropriately generalized to intra-individual relationships between natural environment of PA, recovery experiences and perceived stress. Thus, if both levels of analysis are taken into account, the risk of results being misinterpreted can be minimized. The fact that this has rarely been done in existing studies might be one reason for the inconsistent findings in green exercise research (Thompson Coon et al., 2011; Lahart et al., 2019; Mnich et al., 2019).

Thus, the aim of this study is to examine whether the potential relationship between PA in a natural environment and perceived stress is mediated by recovery experiences, specifically by psychological detachment and relaxation. While recovery experiences in previous studies in this area were recorded retrospectively (e.g., in the evening, Feuerhahn et al., 2014) or in a general manner (Korpela et al., 2014), the present research will use an EMA approach and assess specific recovery experiences directly in the scenario and across multiple bouts of PA in a sample of Swiss working adults. A working population will be examined because work is a main source of stress and to test the role of psychological detachment from work. The present findings have the potential to optimize exercise recommendations to achieve the most beneficial health effects.

## Methods

### Sample

Inclusion criteria for the study were that participants worked at least 80%, were fluent in German, that they were between 20 and 59 years of age and that they engaged in exercise and sport activities sporadically or regularly (at least once a week). Furthermore, individuals were excluded if they were obese (BMI ≥ 30), because body weight might influence (recovery) experiences during PA and should consequently be controlled for (Toft and Uhrenfeldt, 2015). Finally, top athletes were excluded from the study, because the study focused on non-competitive PA behavior. Participant recruitment took place via the health management of more than 20 different service companies in the sectors credit/insurance or information/communication and through personal contacts of study assistants. The final sample consists of 62 individuals, of which 56.5% were female. The average age was *M* = 32.30 years (*SD* = 10.23, range: 22–59 years). Fifty Three percent of participants indicated having a degree from a university or a university of applied science as their highest level of education. The average full-time equivalent participants worked was 95.4%. 35.5% of the participants held a leadership position. Eighty percent of the participants did not have children. 8.1% of the participants reported at the beginning of the study that they habitually exercised <75 min per week and 91.9% exercised 75 min per week or more.

### Study Design and Procedure

Participants who met the inclusion criteria and who were interested in participating in the study were individually invited to a face-to-face meeting or a phone call. There, they received information about the study goals and procedure. All participants gave their written informed consent and were free to decline participation. Afterwards, study assistants handed out a smartphone (Motorola Moto G5) on which the app “movisensXS” (Movisens GmbH, Karlsruhe, Germany) was installed. With this app, questionnaires can be implemented offline and from anywhere. All participants received detailed verbal instructions on how to answer the questionnaires in the app and answered sample items to become familiar with the app.

**Figure 1:**
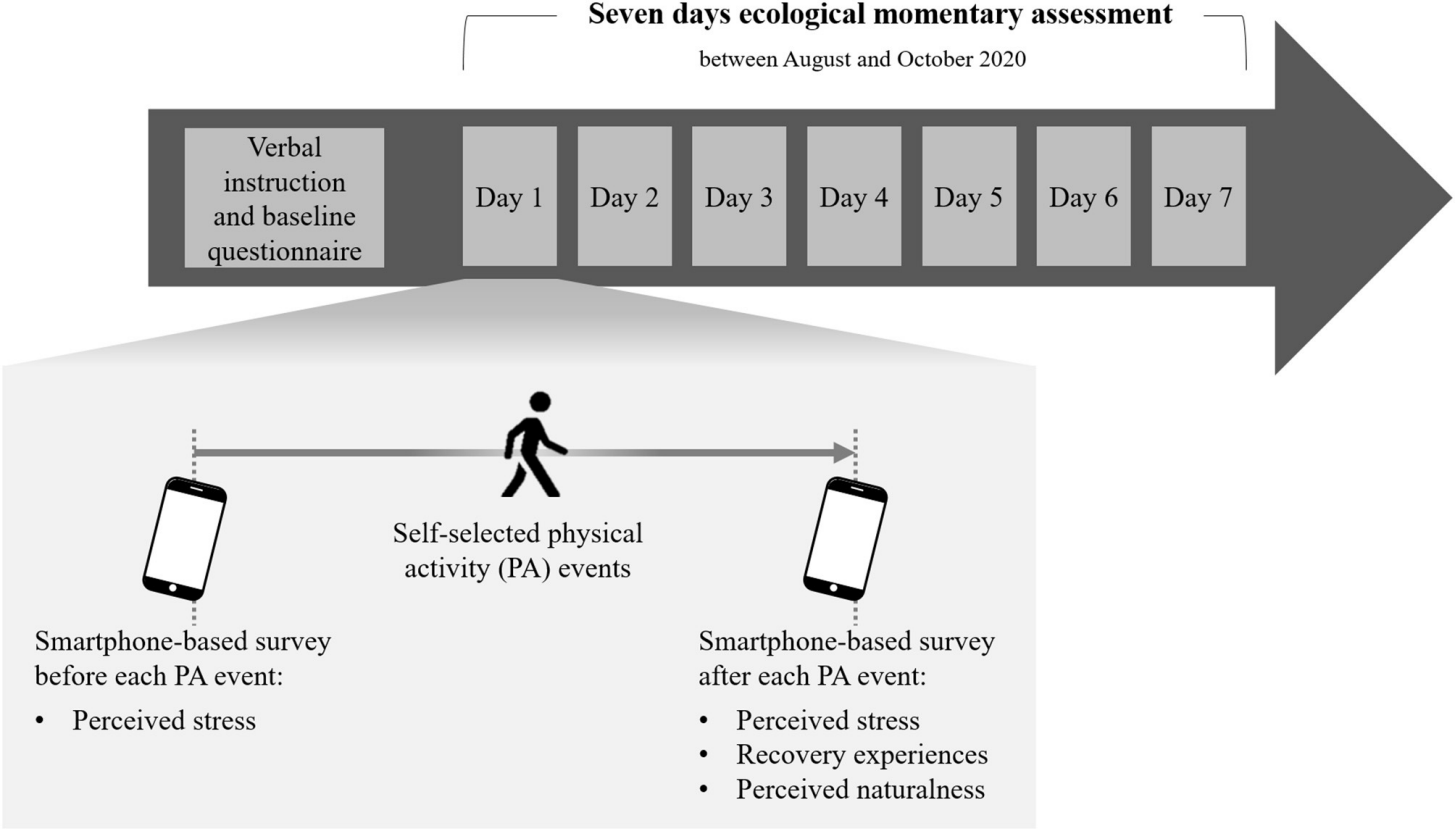
Study procedure.

Basic sociodemographic information (e.g., age) was gathered via questionnaires before starting the EMA procedure. The EMA procedure for each participant lasted 7 days and took place sometime between the beginning of August and the end of October 2020. If participants were involved in PA lasting at least 10 min on a given day, they had to answer questionnaires on the smartphone both prior to the activity and afterwards (see Figure 1). It was emphasized that they needed to fill in the questionnaires as close in time to the PA session as possible (e.g., before showering or changing) and that they should not change their PA behavior because of the study. All participants received a definition of leisure-time PA to make clear when they had to complete the questionnaires (Fuchs et al., 2015).

As compensation, participants received a voucher for a swiss supermarket worth 35 Swiss francs (20 CHF for participation and another 15 CHF for completing seven days). In addition, they received individual feedback on their PA behavior (a summary of their weekly exercise volume and estimated energy consumption) after finishing the study. The Ethics Committee of the University of Bern's Faculty of Human Sciences approved the study.

### Measures

#### Mode and Type of Physical Activity

The mode of PA was assessed after PA using the classification schema of Strath et al. (2013). Participants were asked to indicate whether they had just engaged in (a) exercise or sport in leisure time, (b) physical activity for transport, (c) domestic physical activity, (d) occupational physical activity, or (e) others. Furthermore, individuals had to name the type of PA (e.g., jogging, cycling).

#### Perceived Naturalness

Naturalness was assessed after PA using a visual analog scale by Mackay and Neill (2010). In their quasi-experimental study, they found a negative association between perceived naturalness and post-exercise anxiety, which supports the validity of the scale (see also Klaperski et al., 2019). In the visual analog scale, participants had to indicate the PA environment's degree of naturalness. The scale ranged from 0 “*artificial/urban”* to 100 “*natural.”* A skyline icon was shown at the artificial end of the scale and a forest icon was shown at the natural end of the scale.

#### Recovery Experiences During PA: Psychological Detachment and Relaxation

Psychological detachment and relaxation were assessed after PA with an adapted version of the recovery experience questionnaire (REQ) by Sonnentag and Fritz (2007). In the original REQ people are asked to rate recovery experiences in a general manner (item stem: “In my leisure-time…”). In order to capture recovery experiences situationally and with regard to PA, the original item stem was adapted to “During the PA event I have just carried out… ” Of the original four items per subscale, those two were selected which had the highest loadings in the factor analysis (Sonnentag and Fritz, 2007; Chawla et al., 2020) and which simultaneously seemed appropriate in the PA context. Example items include “I forgot about work” (psychological detachment) and “I kicked back and relaxed” (relaxation). The response format was a 5-point scale from one “*I do not agree at all”* to 5 “*I fully agree.”* The internal consistency of the subscales was rated satisfactory to good (psychological detachment: *r*_within−personlevel_ = 0.77; *r*_between−personlevel_ = 0.98; relaxation: *r*_within−personlevel_ = 0.52; *r*_between−personlevel_ = 0.73).

#### Perceived Stress

Perceived stress was assessed before and after PA with a visual analog scale widely used in research and clinical practice (Lesage et al., 2012). Existing studies have demonstrated its good reliability and sensitivity for assessing perceived stress caused by acutely distressing events. Furthermore, medium to high correlations with other stress questionnaires support the validity of the scale (Lesage et al., 2012). Participants had to indicate how stressed they felt at that moment. The response scale ranged from 0 “*not stressed”* to 100 “*very stressed.”*

### Data Preparation and Analysis

For the analyses, only sport and exercise activities in leisure time (e.g., hiking, soccer, yoga) and transportation related activities (e.g., walking or cycling to work) were considered. We excluded occupational PA, because the present study focuses on non-work PA experiences. Furthermore, we excluded physically demanding off-job duties, such as cleaning, as research shows that they often deplete resources and consequently should not be associated with recovery experiences (Ginoux et al., 2021). A two-level mediation analysis was applied, which allows for the consideration of the hierarchical structure of the data. The multiple measures of naturalness, recovery experiences during PA and perceived stress define the lower level of the hierarchy (level 1). These components were nested within the subjects that defined the higher level of the hierarchy (level 2). Based on theoretical considerations and empirical findings, the model was controlled for perceived stress before PA (Bennett et al., 2018; Sonnentag, 2018a). In total, 62 participants engaged in 423 PA events. The analysis was done with MLmed, a SPSS macro by Hayes and Rockwood (2020). A random-intercept-random-slope model^1^ was calculated and estimated using a full maximum likelihood method. Indirect effects were tested for significance using Monte Carlo confidence intervals. All variables were centered around the group mean. The alpha level of the tests was set to *p* < 0.05.

At the beginning, the entire data set was checked for multivariate outliers with Mahalonobis distance (χ2 at *p* < 0.001; Tabachnick and Fidell, 2013). This led to three PA events being removed from the dataset. The proportion of missing values was 15% (392 of total 2,569 data points). However, as Little's test (Little and Rubin, 2020) was not significant, it could be assumed that values are missing completely at random (χ2 (30) = 27.83, *p* = 0.580). Consequently, missing values were imputed using the expectation-maximation algorithm (Tabachnick and Fidell, 2013).

## Results

### Descriptive Analysis

Data analyses are based on 420 PA events of 62 people. On average, 6.82 PA events per person were taken into account (*SD* = 3.99, range: 2–22). Fifty five percent were exercise and sport activities, such as running or playing football. Fourty five percent were daily activities for transport, such as cycling to work or walking to the grocery store. The average duration of one PA event was 64.21 min (*SD* = 59.76, range: 10 min−420 min).

An overview of the descriptive statistics can be found in Table 1. To see whether study variables varied within individuals across recorded PA events, intra-class correlation coefficients needed to be subtracted from one. Values show that within-person variation of the recovery experiences and perceived naturalness were moderate to high (naturalness: 64%, relaxation: 67%, psychological detachment: 73%). Within-person variation of perceived stress after PA engagement (58%) was slightly lower than before engagement in PA (63%).

**Table 1:** Descriptives of the study variables: Intraclass correlation coefficients (ICC), means *(M)*, variances (σ^2^) and correlations (*p < 0.05).

**Variables [range]**	**ICC**	**Between-person level**						**Within-person level**				
		***M***	**σ^2^**	**Correlations**				**σ^2^**	**Correlations**			
				**2.**	**3.**	**4.**	**5.**		**2.**	**3.**	**4.**	**5.**
1. Perceived naturalness [1–100]	0.361	49.86	1412.26	−0.27	−0.14	0.17	0.07	718.80	−0.21*	−0.18*	−0.02	0.34*
2. Perceived stress_prePA_ [1–100]	0.369	25.88	170.38	–	0.94*	−0.46*	−0.19	279.58	–	0.52*	−0.20*	−0.13*
3. Perceived stress_postPA_ [1–100]	0.425	18.77	131.74	–	–	−0.57*	−0.34*	184.73	–	–	−0.48*	−0.39*
4. Psychological detachment during PA [1–5]	0.261	3.96	0.28	–	–	–	0.56*	0.78	–	–	–	0.38*
5. Relaxation during PA [1–5]	0.332	3.26	0.34	–	–	–	–	0.34	–	–	–	–

### Two-Level Mediation Model

**Figure 2:**
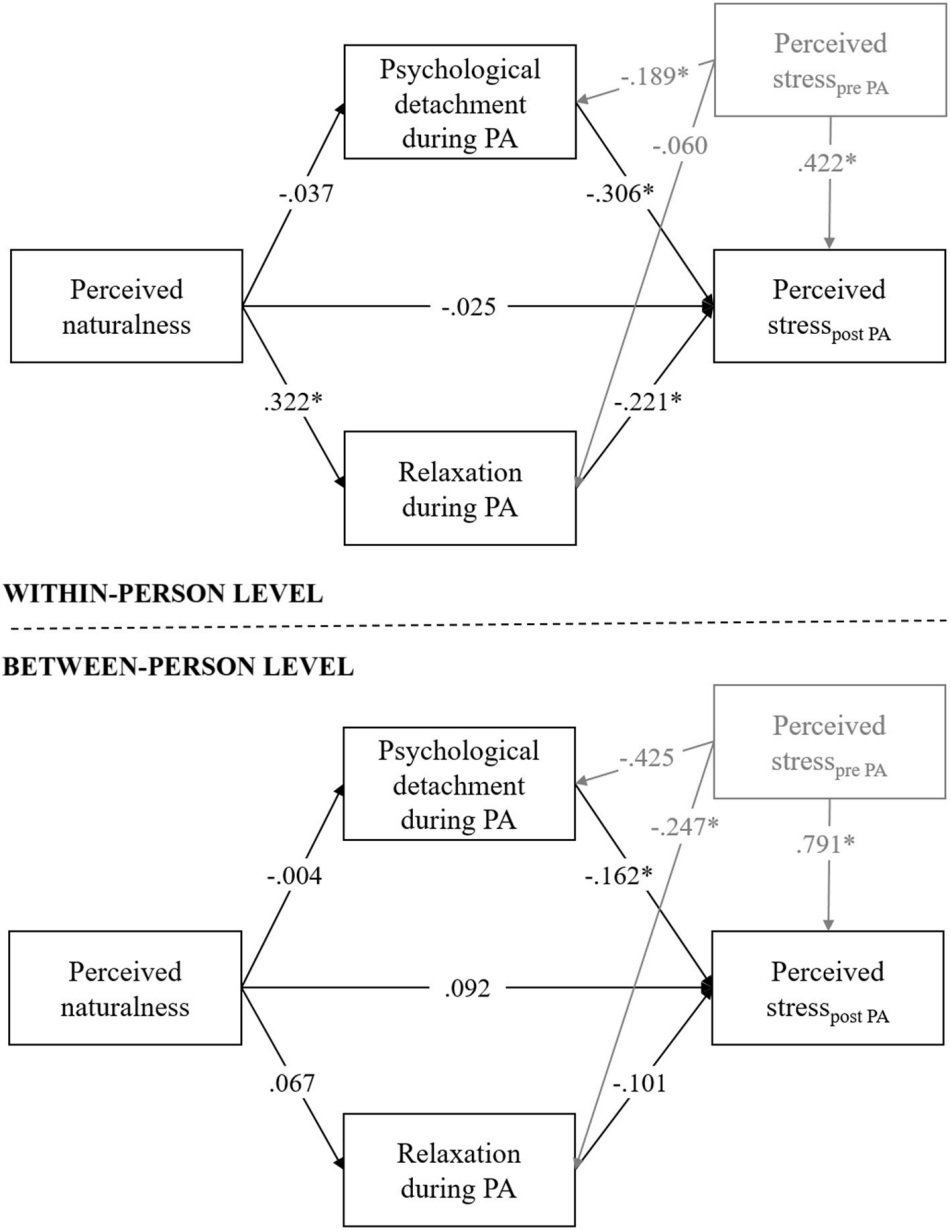
Results of the two-level mediation analysis. All reported regression coefficients (*, *p* < 0.05) are standardized estimates. For a better overview, control variables are shown in gray.

Figure 2 and Table 2 show that the relationships between the study variables differed depending on the level of the model: At the within-person level, perceived naturalness was positively associated with relaxation (β = *0.322, p* < 0.0005) but not with psychological detachment. This result indicates that the more natural a given person perceived the environment during multiple sessions of PA, the more he or she relaxed. In contrast, at the between-person level, perceived naturalness was not associated with either recovery experience. Additionally, at the within-person level, both psychological detachment (β = −0.306, *p* < 0.0005) and relaxation during PA (β = −0.221, *p* < 0.0005) were negatively linked with perceived stress after PA. This means that the more a given individual distanced from work or the more he/she relaxed during multiple sessions of PA, the less stress he or she perceived. On a between-person level, however, only psychological detachment was negatively associated with perceived stress after PA (β = −0.162, *p* = 0.018): Individuals who on average experienced a higher level of detachment during PA perceived less stress after PA.

**Table 2:** Results of the two-level mediation analysis: unstandardized regression coefficients (*, *p* < 0.05), standard errors (SE) with lower and upper bounds of the convidence interval.

***Paths***	***B***	***SE***	***t***	***p***	***Lower bound***	***Upper bound***
Perceived stress_postPA_ intercept	18.84*	6.13	3.07	<0.0005	6.56	31.11
Psychological detachment intercept	4.41*	0.25	17.54	<0.0005	3.91	4.91
Relaxation intercept	3.44*	0.17	20.67	<0.0005	3.12	3.77
*Within-person level*						
Perceived naturalness → Perceived stress_postPA_	−0.01	0.02	−0.55	0.59	−0.06	0.03
Perceived naturalness → Psychological detachment	0.00	0.00	−0.57	0.57	−0.01	0.00
Psychological detachment → Perceived stress_postPA_	−4.72*	0.77	−6.15	<0.0005	−6.27	−3.17
Perceived naturalness → Relaxation	0.01*	0.00	5.08	<0.0005	0.01	0.01
Relaxation → Perceived stress_postPA_	−3.61*	0.75	−4.82	<0.0005	−5.09	−2.14
Perceived stress_prePA_ → Perceived stress_postPA_	0.34*	0.04	8.43	<0.0005	0.26	0.43
Perceived stress_prePA_ → Psychological detachment	−0.01*	0.00	−3.35	<0.0005	−0.02	0.00
Perceived stress_prePA_ → Relaxation	0.00	0.00	−0.95	0.34	−0.01	0.00
*Between-person level*						
Perceived naturalness → Perceived stress_postPA_	0.05	0.03	1.60	0.11	−0.01	0.12
Perceived naturalness → Psychological detachment	0.00	0.00	−0.03	0.97	−0.01	0.01
Psychological detachment → Perceived stress_postPA_	−3.52*	1.38	−2.56	0.01	−6.27	−0.77
Perceived naturalness → Relaxation	0.00	0.00	0.87	0.38	0.00	0.01
Relaxation → Perceived stress_postPA_	−1.98	1.21	−1.64	0.11	−4.39	0.43
Perceived stress_prePA_ → Perceived stress_postPA_	0.70*	0.05	12.72	<0.0005	0.59	0.80
Perceived stress_prePA_ → Psychological detachment	−0.02*	0.01	−3.29	<0.0005	−0.03	−0.01
Perceived stress_prePA_ → Relaxation	−0.01*	0.00	−3.33	<0.0005	−0.02	0.00

Both on the within person- and the between person-levels, no evidence for direct effects were found from perceived naturalness on perceived stress after PA. However, the within person-level model revealed a small indirect effect between perceived naturalness and perceived stress after PA via relaxation. This indirect path reached statistical significance (β = −0.07, *B* = −0.04 [CI 95%: −0.059; −0.018]), while the other indirect pathway did not.

## Discussion

The present study aimed to investigate whether the relationship between perceived naturalness and perceived stress after PA was mediated by specific recovery experiences. This research expands upon previous studies by examining a potential psychological mechanism of green exercise. By investigating psychological detachment and relaxation, we focused on two recovery experiences that have previously only been vaguely addressed in prominent theories in the field of green exercise (Ulrich, 1983; Kaplan and Kaplan, 1989), but that have been intensively researched in occupational health psychology (Bennett et al., 2018). In the present research, self-reports were collected multiple times from individuals, proximal to the time and place PA occurred. Such an EMA approach has recently been identified as promising for future green exercise research (Mnich et al., 2019), because it more likely captures phenomena that vary over time or space compared to traditional cross-sectional, retrospective and summary methods.

As hypothesized, the relaxation as a recovery experience served as mediator in this study, but only on a within-person level. The more natural a given individual rated the PA setting, the more relaxed he or she felt during PA. In turn, the more relaxed the individual was, the less stress he or she perceived after PA. However, in contrast to our assumptions, psychological detachment did not serve as mediator, neither at the within- nor the between-person levels. Overall, the study shows that the associations between the study variables was different depending on which level of analysis was being considered. An indirect effect of perceived naturalness on perceived stress after PA via relaxation could only be observed on a within-person level, where multiple PA events are considered as experienced within an individual. Thus, it seems to be a situational mechanism rather than a general one.

Our study results did not support Kaplan and Kaplan's (1989) assumption that vegetation-rich settings create a sense of “being away,” which mediates the effect of naturalness on well-being. In line with the meta-analytic findings of Bennett et al. (2018), psychological detachment was associated with low stress after PA. However, psychological detachment was not linked to the naturalness of the environment of PA at either level of analysis. This finding might merely imply that the engagement in PA behavior is relevant to achieve detachment from work (Feuerhahn et al., 2014), and that there is no additional gain from a vegetation-rich environment. This would mean that taking an active “time-out” from work is in itself sufficient to recover from stress, a conclusion which has also been drawn in previous studies (Bahrke and Morgan, 1978). It is also possible that it is not necessarily the PA *setting* that influences psychological detachment, but rather more so the activity *type*. Fuchs and Klaperski (2018), for instance, suggest that activities which require being in the “here and now” as well as a deep involvement in the current action (e.g., climbing or game sports) prevent rumination or worry about hassles and therefore allow for mental distance from work. This assumption is supported by a recent quasi-experimental study in which it was demonstrated that bouldering facilitated mindfulness (e.g., being present) more when compared to a fitness training session (Wheatley, 2021).

### Limitations and Future Directions

There are some limitations in this study that can lead to future research opportunities. Firstly, participants were mostly active, middle-aged and well-educated workers from the service sector. This relatively homogeneous sample limits the generalizability of the findings. Future research should explore whether the psychological mechanism observed in this study also appears in other occupational groups and professional contexts (Sonnentag et al., 2017). For instance, it can be assumed that people with physically demanding outdoor jobs (e.g., gardeners) experience less relaxation during leisure time PA in a natural environment than people with more cognitively demanding indoor jobs (e.g., administrative employees). Assessing different types of pre- and post-exercise recovery states and detachment (e.g., emotional, cognitive, or physical) could shed more light on these type of matching questions (see also Balk et al., 2017). Secondly, this study focused on perceived stress as an important determinant for well-being, yet well-being itself was not assessed. Although the amount of perceived stress seems to be particularly relevant in the work setting, future studies may assess affective well-being based on a dimensional approach (e.g., vigor and fatigue; Bennett et al., 2018). This would make it easier to compare the findings with existing research from the field of green exercise and occupational health psychology. Thirdly, the present research was limited to two specific recovery experiences as mediators. Psychological detachment and relaxation were chosen based on key assumptions of the ART, SRT (Ulrich, 1983; Kaplan and Kaplan, 1989) and on existing research in the field of occupational health psychology (Sonnentag and Fritz, 2007; Bennett et al., 2018). However, future research should investigate the impact of further mediators, but also moderators. An additional mediator might be, for example, “mind wandering.” Mind wandering is defined as thinking unrelated to the ongoing task (Smallwood and Schooler, 2015) and mentally moving hither and thither without a fixed course or certain aim (Christoff et al., 2016). One can hypothesize that subtly fascinating natural environments (e.g., clouds) support mind wandering (Kaplan and Kaplan, 1989; Williams et al., 2018) and this in turn reduces stress (Miś and Kowalczyk, 2020). Potential moderators may be an individual's habitual environment, and representations and expectations of green exercise. A person who lives and works in an urban neighborhood could experience physical exercise in green surroundings as well as the naturalness of an environment differently than a person living and working in a rural environment. Green exercise could be experienced as a strong contrast and therefore enhance the effect of PA on stress, possibly also based on neurological differences (e.g., Lederbogen et al., 2011). Furthermore, an individual's goals for PA (e.g., Schmid et al., 2018) could be considered as possible moderators, as studies showed that such motivational aspects influence the effect of PA on affective well-being (e.g., Jeckel and Sudeck, 2018). Fourthly, data collection in this study took place over three months. While some participants took part during more summer-like weather conditions in August, other participants had more fall-like conditions in October. These different seasons (different temperatures, colors) may have influenced the findings. In line with this, Bloom et al. (2017) found that the experience of detachment and relaxation during a park walk differed in spring and fall. Fifth, the present analyses are based solely on self-reported data. Ideally, future studies should assess leisure-time PA and its natural environment in an objective way (e.g., with accelerometers and portable global positioning system data). Such complementary analyses might help to get a deeper understanding of PA's impact on stress and recovery. Finally, it cannot be ruled out that the measure of perceived stress before PA was already influenced by natural settings. It is possible, for example, that people were already impacted by rich vegetation while being on their way to green space. Research shows that even a short view of natural elements can provide psychological benefits (Lee et al., 2015).

## Conclusion

This study provides new insights into the psychological mechanisms which might underlie the positive effects green exercise has on stress levels and mental well-being. It shows that being active in natural settings further increases the benefits of PA on perceived stress, by altering relaxation on a within-person level. Thus, being active within a vegetation-rich environment may be an effective way to calm down. Overall, the current findings suggest that research should put greater emphasis on examining the specific psychological mechanisms of green exercise to make even better use of its beneficial effects in the future. Furthermore, result patterns highlight the importance of using a multilevel approach in green exercise research.

## Data Availability Statement

The raw data supporting the conclusions of this article will be made available by the authors, without undue reservation.

## Ethics Statement

The studies involving human participants were reviewed and approved by The Ethics Committee of the University of Bern's Faculty of Human Sciences. The patients/participants provided their written informed consent to participate in this study.

## Author Contributions

JS, SK, and GS contributed to the conception and design of the study. JS and LI acquired the data. JS and LI performed statistical analysis. JS wrote and drafted the manuscript. JS, LI, SK, and GS contributed to data analysis and interpretation. JS, LI, SK, and GS critically reviewed initial versions of the manuscript. All authors revised the manuscript critically for important intellectual content, as well as read and approved the final manuscript. All authors contributed to the article and approved the submitted version.

## Conflict of Interest

The authors declare that the research was conducted in the absence of any commercial or financial relationships that could be construed as a potential conflict of interest.

## Publisher's Note

All claims expressed in this article are solely those of the authors and do not necessarily represent those of their affiliated organizations, or those of the publisher, the editors and the reviewers. Any product that may be evaluated in this article, or claim that may be made by its manufacturer, is not guaranteed or endorsed by the publisher.

## Footnotes

^1^Although slopes were principally specified as random effects in the model, four paths had to be fixed due to estimation problems. This applied to the following paths: relaxation → stress_postPA_; naturalness → relaxation; stress_prePA_ → relaxation (see Figure 2).
